# Assessment of the prozone effect in malaria rapid diagnostic tests

**DOI:** 10.1186/1475-2875-8-271

**Published:** 2009-11-30

**Authors:** Philippe Gillet, Marcella Mori, Marjan Van Esbroeck, Jef Van den Ende, Jan Jacobs

**Affiliations:** 1 Department of Clinical Sciences, Institute of Tropical Medicine (ITM), Nationalestraat 155, B 2000 Antwerp, Belgium; 2 Faculty of Health, Medicine and Life Sciences (FHML), Maastricht, The Netherlands

## Abstract

**Background:**

The prozone effect (or high doses-hook phenomenon) consists of false-negative or false-low results in immunological tests, due to an excess of either antigens or antibodies. Although frequently cited as a cause of false-negative results in malaria rapid diagnostic tests (RDTs), especially at high parasite densities of *Plasmodium falciparum*, it has been poorly documented. In this study, a panel of malaria RDTs was challenged with clinical samples with *P. falciparum *hyperparasitaemia (> 5% infected red blood cells).

**Methods:**

Twenty-two RDT brands were tested with seven samples, both undiluted and upon 10 ×, 50 × and 100 × dilutions in NaCl 0.9%. The *P. falciparum *targets included histidine-rich protein-2 (HRP-2, n = 17) and *P. falciparum*-specific parasite lactate dehydrogenase (Pf-pLDH, n = 5). Test lines intensities were recorded in the following categories: negative, faint, weak, medium or strong. The prozone effect was defined as an increase in test line intensity of at least one category after dilution, if observed upon duplicate testing and by two readers.

**Results:**

Sixteen of the 17 HRP-2 based RDTs were affected by prozone: the prozone effect was observed in at least one RDT sample/brand combination for 16/17 HRP-2 based RDTs in 6/7 samples, but not for any of the Pf-pLDH tests. The HRP-2 line intensities of the undiluted sample/brand combinations with prozone effect (n = 51) included a single negative (1.9%) and 29 faint and weak readings (56.9%). The other target lens (*P. vivax*-pLDH, pan-specific pLDH and aldolase) did not show a prozone effect.

**Conclusion:**

This study confirms the prozone effect as a cause of false-negative HRP-2 RDTs in samples with hyperparasitaemia.

## Background

Malaria rapid diagnostic tests (RDTs) are lateral flow immunochromatographic tests that detect *Plasmodium *antigens by antibody-antigen interactions on a nitrocellulose test strip. Capillary or venous blood and a lysis buffer are added to the strip: if present in the sample, the *Plasmodium *antigen is bound to a detection antibody. This detection antibody is usually a monoclonal mouse-antibody conjugated to a signal, mostly colloidal gold. The antigen-detection antibody-conjugate complex diffuses further across the strip until it is bound to a second antibody: this so-called capture antibody reacts to another epitope of the target antigen. As the capture antibody is fixed on a narrow section of the strip, the conjugated signal is concentrated and becomes visible as a cherry-red or purple colored line. The excess of detection antibody-conjugate that was not bound by the antigen and the capture antibody moves further until it is bound to a goat anti-mouse antibody, thereby generating a control line. The *Plasmodium *antigens targeted by RDTs include those specific to *Plasmodium falciparum *(histidine-rich protein-2 (HRP-2) and *P. falciparum*-specific parasite lactate dehydrogenase (Pf-pLDH)), the antigen specific to *Plasmodium vivax *(*P. vivax*-specific parasite lactate dehydrogenase, Pv-pLDH) and the antigens common to *P. falciparum*, *P. vivax*, *Plasmodium ovale *and *Plasmodium malariae *(pan-species parasite lactate dehydrogenase (pan-pLDH) and aldolase). The RDT strip is produced either as a simple dipstick or fixed in a cassette or cardboard format. RDTs combine a control line with one or more antigen detecting test lines: those with a single test line are named two band RDTs, those with two and three antigen test lines are known as three-band and four-band RDTs respectively.

The use of malaria rapid diagnostic tests is expanding, both in endemic and non-endemic settings [1]. In 2007, more than 70 million RDTs have been procured [2] and in many endemic countries, RDTs are now being rolled out as the instrument of choice for parasite-based malaria diagnosis and patient management at all levels of health care [3]. Under controlled conditions, RDTs have shown sensitivities close to 100% for the detection of *P. falciparum*, the most life-threatening species. However, there are still false-negative results: most of them occur at low parasite densities (< 100 asexual parasites/μl or < 0.002% of red blood cells infected), but others occur at high parasite densities, in particular at hyperparasitaemia, defined by the World Health Organization (WHO) as infections with > 5% of red blood cells infected [4]. Mostly, the latter are ascribed to genetic variations of the HRP-2 [3-5], but the prozone effect is also cited as an explanation [1,6-10]. The prozone effect (also known as high dose-hook phenomenon) is defined as false-negative or false-low results in immunological reactions, due to an excess of either antigens or antibodies [11]. It occurs particularly in one-step immunoassays, such as agglutination tests, for which serial dilutions are advised to trace the effect [12]. In the case of hyperparasitaemia in RDTs, high antigen concentrations will block all available binding sites of both the detection and the capture antibodies, thereby preventing the binding of the antigen-detection antibody-conjugate complex to the capture antibody, with failure of signal generation. Simple dilution of the sample will correct this effect. Despite frequently cited in literature on RDTs, there is only a single original report in which the prozone effect in RDT is unequivocally demonstrated by appearance of the test line upon dilution of the sample [13]. Since attention was drawn to this effect by a recent case (see below) presented at the Institute of Tropical Medicine (ITM), a panel of RDTs was challenged to clinical samples with *P. falciparum *hyperparasitaemia.

### Case description

An EDTA-blood sample of a 69-year old male patient, returning from Nigeria was submitted to the reference laboratory of ITM. The referring laboratory had made the microscopic diagnosis of *P. falciparum *malaria with a parasite density of 27.5%. However, they were puzzled about the result of the RDT, which they performed in conjunction to microscopy: in addition to the control line, the test showed a clear pan-*Plasmodium *aldolase line but there was no HRP-2 line visible. According to the instructions of the manufacturer, this combination points to the diagnosis of non-falciparum malaria.

Upon receipt of the sample, the diagnosis of *P. falciparum *was confirmed as well as the parasite density. The sample was tested with the usual panel of RDTs used in ITM. The BinaxNow^® ^(Binax, Scarborough, Maine, U.S.) and the SD FK60 Malaria Ag *P. falciparum*/Pan (Standard Diagnostics, Hagal-Dong, Korea) showed faint and barely distinguishable HRP-2 lines in combination with clear control and pan-*Plasmodium *lines (aldolase and pan-pLDH respectively). The Optimal-IT test (DiaMed AG, Cressier s/Morat, Switzerland) showed clear lines for both Pf-pLDH and pan-pLDH. A 10 × dilution of the EDTA-blood in NaCl 0.9% resulted for both the BinaxNow^® ^and the SD FK60 tests in clearly distinguishable HRP-2 lines of intensities equal to the control line. When doubled volume of the undiluted blood was applied to the sample pad, the HRP-2 line was still visible as a faint line in the SD FK60, but was no longer visible in the BinaxNow^® ^test. It was concluded that the prozone effect was the cause of the false-low and false-negative test line intensities.

## Methods

### Patients' samples

In this study EDTA-blood samples obtained from international travellers presenting at the outpatient clinic of ITM, Antwerp, Belgium, as well as samples submitted by Belgian laboratories to ITM in its functions as the National Reference Centre were used. Part of them were fresh samples, the other samples had been stored at -70°C till analysis. For all samples, the diagnosis of malaria was made by microscopy and confirmed with species-specific PCR as previously described [14]. Parasite density was assessed by counting the number of asexual parasites against 200 white blood cells in a thick film, and converting this value to parasites/μl using the actual count or the standard of 8,000 white blood cell/μl [15]. For the purpose of this study, the more convenient parasite density as expressed in % of infected red blood cells was applied, thereby assuming 50,000/μl to be equal to 1% of red blood cells parasitized [15]. *P. falciparum*-infected samples with hyperparasitaemia were selected and a sample with parasite density of 0.1% (5,000/μl) was used as a control sample.

### Malaria rapid diagnostic tests

Malaria RDTs chosen were those included in the WHO list of RDT manufacturers with adequate evidence of good manufacturing practice available online [16] and assessment was focused on those products available in cassette and folded card box format. In addition to this list, other RDTs available on the international market were randomly included. In view of the wide lot-to-lot variations and the ever changing composition of RDTs, it was decided not to display the individual RDT brand names, in line with a similar study that compared RDT heat stabilities [17].

Tests were performed according to the instructions of the manufacturer, except that samples were loaded with a pipette (Finnpipette, Helsinki, Finland) instead of the transfer device supplied by the manufacturer. In case the control line did not appear the result was interpreted as invalid and the test was repeated. In order to qualify the test line intensities, a scoring system of five categories was used: none (no line visible), faint (barely visible line), weak (paler than the control line), medium (equal to the control line) or strong (stronger than the control line) [14]. All tests were carried out in duplicate. Readings were performed by two readers at daylight assisted by a standard electric bulb, and within and not beyond the prescribed delay after application of the sample and buffer. Tests were performed on undiluted samples as well as on samples diluted 10 ×, 50 × or 100 × in saline solution (NaCl) 0.9%.

### Test outcomes and definitions

For the results of test line intensities, consensus readings were considered, *i.e*. the line intensities most frequently scored in the duplicate test/two readers' combination. The prozone effect was defined as an increase in test line intensity of at least one category after dilution, if observed upon duplicate testing and by two readers.

### Statistical analysis

Inter-reader reliability for line intensities was calculated as percentage agreements and kappa values. Reproducibility was expressed as the consistency of line intensity readings for both readers and upon repeating the tests.

### Ethical review

The study was reviewed and approved by the Institutional Review Board of ITM and by the Ethical Committee of Antwerp University, Belgium.

## Results

### Collection of samples and RDTs

Seven samples with hyperparasitaemia were elected, their parasite densities are listed in Additional file 1. Six samples had been stored at -70°C for a period of three to 96 months, one was assessed freshly. They were all obtained in patients returning from sub-Saharan Africa. Twenty-two brands of RDTs were collected, 17 of them targeted *P. falciparum *by detecting HRP-2, the other five detected Pf-pLDH (two of which produced by the same company). The RDT brands included three two-band tests, 15 three-band tests and four four-band tests. The antigens targeting all four common species comprised pan-pLDH (n = 16) and aldolase (n = 2). Fourteen RDTs had CE mark compliance, 14 were included in the WHO list and one is authorized for use in the United States (US FDA approved).

### Inter-reader reliability and reproducibility

Inter-reader reliability for *P. falciparum *test line intensities was high, with 86.7% agreement and a kappa value of 0.79, and discrepancies limited to one category of line intensity (*e.g*. line intensity read as weak by reader 1 and as medium (but not strong) by reader 2). Upon duplicate testing, 82.5% and 80.3% of 319 *P. falciparum *line intensities were identically read by each of both readers respectively. For the non-*falciparum *test line intensities (pan-pLDH and aldolase), agreement and kappa value for line intensities between readers was 88.2% and 0.84 respectively. These differences had no effect on the numbers of samples with prozone effect. All 51 sample/brand combinations, (six samples for 12 brands) with prozone effect showed the effect as defined for both observers, and no additional cases were suggested by the observation of only a single observer.

### Prozone effect

For the Pv-pLDH, pan-pLDH and aldolase lines, there were 136 sample/brand combinations tested: in 51 (36.7%) of them (representing 11/14 RDTs), the 10 × diluted samples showed weaker line intensities as compared to the undiluted samples. The control lines were well visible in all cases, except in four invalid brand/sample combinations for a single brand (RDT nr. 4, Additional file 1). For the *P. falciparum *lines, there was a clear difference between HRP-2 lines and Pf-pLDH lines. For the control sample of parasite density of 0.1%, all but one RDTs showed medium or strong line intensities upon undiluted testing; RDT nr.12 showed a weak HRP-2 line intensity. When assessed with the samples with hyperparasitaemia, the five three-band tests targeting Pf-pLDH did not show a prozone effect. For one RDT, there were two samples that showed a decrease in Pf-pLDH line intensity at the 10 × dilution; the other combinations did not change line intensity upon dilution. By contrast, the prozone effect was observed for at least one sample in all, but one HRP-2 RDT brands (Additional file 1). The single RDT brand that did not display a prozone effect was RDT nr. 12, which showed a decrease in test line intensity upon 10 × dilution. The prozone effect tended to occur more frequently in particular brands and samples and was not directly related to the parasite count: for instance, the sample with 11.6% parasite density showed the prozone effect for all but one brand, whereas the sample with 35.0% parasite density did so for 8/21 brands tested. There was no difference in prozone effect between RDTs that were CE-marked, FDA approved or WHO-listed and those that were not. In terms of test line intensities, the distribution for the undiluted sample/brand combinations with prozone effect (n = 51) was as follows: a single (1.9%) negative reading, four (7.8%) faint readings, three (5.9%) either faint or weak readings (depending on the observer), 22 (43.1%) weak readings and 21 (41.2%) medium readings. This means that for a total of 29 (56.9%) of sample/brand combinations (in 12/17 RDTs) with the prozone effect, the undiluted sample showed a faint or weak HRP-2 line. Maximum (strong) line intensities were obtained at a 10 × dilution for 28 out of 51 sample/brand combinations. For the remaining 23 combinations there was enough additional material for 16 combinations to perform 50 × or 100 × dilutions, in which 12 and four reached respectively the strong line intensity.

## Discussion

RDTs offer great potential for the timely and accurate diagnosis of malaria, thereby leading to prompt and appropriate treatment. They have found their place in both malaria-endemic and non-endemic settings. In endemic settings, they offer parasite-based diagnosis in the absence of competent laboratory infrastructures as they can be carried out by non-specialized health care workers [18,19]. In non-endemic settings, where microscopic expertise is lacking due to low incidence, they are used as adjunct to microscopy especially outside office hours but also as bedside point of care tests [20,21]. In addition, RDTs are marketed for self-use by travellers [10]. In this study, RDTs were challenged with a panel of clinical samples with *P. falciparum *hyperparasitaemia. The prozone effect was observed for the HRP-2 test lines in 16/17 RDTs, and was consistent among the two observers. None of the *P. falciparum *specific Pf-LDH lines (tested in five RDTs) showed the prozone effect, nor did any of the pLDH and aldolase lines.

The present study has its limitations. A calibrated pipette was used instead of the manufacturer's transfer device, thereby bypassing a possible error of the kit's application system. Next, in reference to the original report [13], dilutions were made in NaCl 0.9% and not, for instance, in the kit's diluent. Also, this evaluation was performed in a reference setting, with expert technicians who are used to evaluate RDTs and who are trained not to disregard faint positive lines, thereby possibly underestimating the incidence of the prozone effect as compared to field settings. Despite these limitations, this study documents the prozone effect among the present panel of RDTs according to stringent and reproducible criteria.

Most reviews and leading authorities point to the possibility of the prozone effect in RDTs [1,8,22,23], but there is only a single original report describing this effect in a RDT: in 1999, Risch and co-workers described a patient returning from Yemen, with a *P. falciparum *infection at a parasite density of 30%. The RDT they used (ICT Malaria, Pf, ICT, Australia - a HRP-2 targeted two band test which is no longer marketed) showed no test line for the undiluted sample, but a clearly distinguishable line at 10 × dilution in NaCl 0.9% [13]. Another report described, as part of a prospective study, a patient returning from The Gambia, with microscopic diagnosis of *P. falciparum *at a parasite density of 31%. For the BinaxNow^® ^Malaria Pf/Pv test, the authors reported observations identical to those presently described, *i.e*. a faint HRP-2 line but a strong *P. vivax*-test line (the latter line representing in fact pan-*Plasmodium *LDH reactivity). Although the authors described this effect in full detail and added a picture, they did not refer to the possibility of the prozone effect and did not carry out dilution studies. In addition to these reports on hyperparasitaemia there are rare but consistent reports of unexplained failures of mainly HRP-2 RDTs at parasite densities in the intermediate ranges (*e.g*. between 10,000/μl and 100,000/μl (0.2% and 2% respectively [1,9,12,14,24-31]). The most frequently cited explanation for these failures is the presence of HRP-2 polymorphisms [1,3,5,6,22], although the polymorphisms that are less likely to be picked up by RDTs are geographically confined to the Asia-Pacific region whereas many of the failures occurred in field settings in Africa [5]. The prozone effect in these samples can be an alternative explanation, but at present samples with intermediate parasite densities were not included in this study.

The observation that, among the presently studied RDTs, HRP-2 brands, but not Pf-pLDH brands are subject to the prozone effect is of interest but remains unexplained. Compared to the Pf-pLDH based RDTs, HRP-2 based RDTs also tend to be more affected by the rheumatoid factor, giving rise to false-positive results [32,33]. In addition, the absence of the prozone effect in the pan-pLDH and aldolase lines is in line with this observation. Although the prozone effect was observed in none of the currently tested Pf-pLDH based RDTs, further research should be done to confirm the absence of the prozone effect in other Pf-pLDH RDTs. It is also of note that the prozone effect did not occur in clear relation to the parasite density: this may be due to different factors affecting the circulating HRP-2 concentration, such as capillary sequestration of the parasites, variations in the production of antigen production during the cycle as well as by strain differences [34,35].

In the present study, only a single sample/brand combination showed complete absence of HRP-2 test line and four samples showed faint line intensities, representing only 5/51 (9.8%) of the tests affected by prozone. However, three additional tests were read as faint by one out of two observers. Further, it should be stressed that the present readings were made by experienced technicians who were trained to interpret faint, weak and medium tests lines, and who were working in reference conditions. By contrast, misinterpretation of faint lines as negative results is a common mistake made by inexperienced staff, travellers and community health care workers both in endemic and non-endemic settings [18,19,21,36] In endemic settings, readings of RDTs are frequently performed in unfavorable light conditions during evening and night shifts [37]. Finally it should be noted that the four faint lines all occurred in two of the three two-band RDTs that are frequently used in field setting.

The consequences of a falsely negative interpretation are serious: in the case of two-band tests, the diagnosis of malaria may be missed, and in the case of a three-band test, an infection with *P. falciparum *will be erroneously diagnosed as a non-falciparum species. Of note is the observation with the submitted sample described in the case report: when the double sample volume was applied, there was complete absence of the HRP-2 test line in one of the RDTs. This may also cause problems in field settings, where there is a tendency to apply more than the required sample volume [38,39].

Although among the presently challenged panel the prozone effect was common, it is yet unclear how frequent it occurs in routine diagnosis. As to the use of RDTs in low resource settings, it is of note that all three tested HRP-2 two-band brands are used in high numbers by non-governmental organizations in emergency relief operations and are presently introduced in national malaria control programs [3]. For example, the 3,000,000 RDT tests provided in 2008 by the Global Fund to Fight AIDS, Tuberculosis and Malaria, all belonged to these three brands [40]. The impact of prozone for these three HRP-2 two-band brands is of concern as it occurred in two, four and six samples respectively. Moreover, the test line intensities obtained with undiluted samples were faint or weak in four and six samples respectively.

In setting of ITM, samples with hyperparasitaemia occurred at a frequency of 5.5% among 200 single-patient *P. falciparum *samples processed over a 23-month period (May 2007-April 2009). Further study, including incident reporting and large-scale prospective testing, should be performed both in the endemic and the non-endemic settings in order to have reliable estimates of the prozone effect as a cause of false-negative RDT results.

False-negative results at low parasite densities can be countered by diagnostic algorithms prescribing repeat or serial testing when malaria is suspected and the initial RDT test result is negative [6,12]. By contrast, false-negative results in hyperparasitaemia are to be corrected immediately. What can be done to prevent or overcome the prozone effect? First, it is imperative to train end-users of RDTs in correctly reading and interpreting faint and weak test lines and emphasizing that the appearance of the control line does not guarantee control of all aspects of RDT test performance. Second, for non-endemic settings, previous recommendations to perform competent microscopy in parallel to the RDTs should be emphasized [1]: an instructed lab technician, even when not expert in malaria and not able to perform species identification, is expected to find and recognize *Plasmodium *parasites in samples with hyperparasitaemia and even in samples with lower parasite densities. If microscopy is not possible or feasible, one could consider having a RDT targeting Pf-pLDH at the hand, thereby assuming that Pf-pLDH tests are not prone to the prozone effect. Suspected samples should be retested on a 10 × and if needed a subsequent 50 × dilution of the sample, with dilutions made in NaCl 0.9%, pending further research on the most appropriate diluents. On the regulatory level, it would be interesting to test all marketed RDTs for their susceptibility to the prozone effect.

## Conclusion

In conclusion, the study demonstrated the prozone effect for the detection of *P. falciparum *in 16/17 HRP-2 based RDTs. The effect did not occur in the five Pf-pLDH RDTs neither in the pan-pLDH and aldolase lines. The HRP-2 line intensities in undiluted samples were negative in a single sample/brand combination and displayed faint or weak line intensities in nearly 60% of sample/brand combinations; therefore, the prozone effect is expected to have consequences in diagnosis and patient care both in endemic and non-endemic settings. Dilutions of 10 × and if needed 50 × should be made to detect this effect, and microscopy and/or a Pf-pLDH RDT can be done when the prozone effect is suspected. Further research should confirm the absence of the prozone effect in other Pf-pLDH targeted RDTs and assess the incidence of the prozone effect in false-negative results in both endemic and non-endemic settings.

## Abbreviations

Ag: Antigen; CE: Conformité Européenne; EDTA: Ethylene diamine tetra-acetic acid; FDA: Food and drug administration; FHML: Faculty of Health Medicine and Life Sciences, Maastricht, The Netherlands; HRP-2: histidine-rich protein-2; ITM: Institute of Tropical Medicine; NaCl: Sodium chloride; P.: *Plasmodium*; Pf: *Plasmodium falciparum; *Pv: *Plasmodium vivax; *PCR: polymerase chain reaction; pan-pLDH: pan species parasite lactate dehydrogenase; Pf-pLDH: *Plasmodium falciparum*-specific parasite lactate dehydrogenase; pLDH: parasite lactate dehydrogenase; Pv-pLDH: *Plasmodium vivax*-specific parasite lactate dehydrogenase; RDT: Rapid diagnostic test; WHO: World Health Organization.

## Competing interests

The authors declare that they have no competing interests.

## Authors' contributions

PG and JJ designed the study protocol. MvE and JVDE organized prospective sample collection. MM and PG carried out the test evaluations, and PG performed statistical analysis. PG, MM and JJ analyzed and interpreted the results and drafted the manuscript. All authors contributed to the discussion of the results and the redaction of the manuscript, they all approved the final manuscript.

## Supplementary Material

Additional file 1 **Prozone effect in 17 HRP-2 based malaria rapid diagnostic tests**. Prozone effect was defined as an increase in HRP-2 line intensity upon dilution of the sample as observed in two tests and read by two observers. In case of prozone effect, line intensities for the undiluted samples are recorded in the Table, they are intensities are categorized as **N**egative, **F**aint, **W**eak, **M**edium and **S**trong. For test line intensities, consensus readings of duplicate test read by two readers were considered. If no consensus was reached, both categories are listed. Click here for file
