# Supplementary material for: Assessment of the prozone effect in malaria rapid diagnostic tests
Source: Malar J. 2009 Nov 30;8:271. doi: 10.1186/1475-2875-8-271 (PMC2789093; doi:10.1186/1475-2875-8-271)
Supplement: Additional file 1 — Prozone effect in 17 HRP-2 based malaria rapid diagnostic tests. Prozone effect was defined as an increase in HRP-2 line intensity upon dilution of the sample as observed in two tests and read by two observers. In case of prozone effect, line intensities for the undiluted samples are recorded in the Table, they are intensities are categorized as Negative, Faint, Weak, Medium and Strong. For test line intensities, consensus readings of duplicate test read by two readers were considered. If no consensus was reached, both categories are listed. [file 1475-2875-8-271-S1.doc]

| **Additional file 1: Prozone effect in 17 HRP-2 based malaria rapid diagnostic tests.** Prozone effect was defined as an increase in HRP-2 line intensity upon dilution of the sample as observed in two tests and read by two observers. In case of prozone effect, line intensities for the undiluted samples are recorded in the Table, they are categorized as Negative, Faint, Weak, Medium and Strong. For test line intensities, consensus readings of duplicate test read by two readers were considered. If no consensus was reached, both categories are listed. | | | | | | | | | | | | | | | | |
| --- | --- | --- | --- | --- | --- | --- | --- | --- | --- | --- | --- | --- | --- | --- | --- | --- |
| **Malaria RDT** | | |  | **Samples with hyperparasitemia and origin** | | | | | | | | | | | | |
|  |  |  |  |  |  |  |  |  |  |  |  |  |  |  |  |  |
|  |  |  |  | 5.5 % |  | 6.3 % |  | 9.0 % |  | 11.6 % |  | 17% |  | 27.5 % |  | 35.0 % |
| Nr |  | Type (other antigen detection) |  | Ivory Cost |  | DRC |  | Tanzania |  | Togo |  | Nigeria |  | Nigeria |  | Benin |
|  |  |  |  |  |  |  |  |  |  |  |  |  |  |  |  |  |
| 1 |  | Two-band |  |  |  |  |  |  |  | M |  |  |  | M |  |  |
| 2 |  | Two-band |  | F^a^ |  |  |  |  |  | W |  |  |  | W^a^ |  | W^a^ |
| 3 |  | Two-band |  | W^a,b^ |  |  |  | F^a,b^ |  | F^a,b^ |  | W^a^ |  | F |  | W^a,b^ |
| 4 |  | Three-band (aldolase) |  |  |  | Invalid |  | Invalid |  | N^a^ |  | Invalid |  | Invalid |  | W |
| 5 |  | Three-band (aldolase) |  | M |  |  |  | M |  | W^a,b^ |  |  |  |  |  | W^a,b^ |
| 6 |  | Three-band (pan-pLDH) |  |  |  |  |  |  |  | W^a,b^ |  | M |  | W^a,b^ |  |  |
| 7 |  | Three-band (pan-pLDH) |  |  |  |  |  |  |  | M |  |  |  | M |  |  |
| 8 |  | Three-band (pan-pLDH) |  |  |  |  |  | M |  | W |  |  |  | W ^a,b^ |  | M |
| 9 |  | Three-band (pan-pLDH) |  |  |  |  |  | W |  | W^a,b^ |  | M |  | W^a^/F^a^ |  | M |
| 10 |  | Three-band (pan-pLDH) |  |  |  |  |  | M |  | W^a,b^ |  | M |  | W^a^/F^a^ |  | W^a,b^ |
| 11 |  | Three-band (pan-pLDH) |  |  |  |  |  |  |  | M |  |  |  | M |  |  |
| 12 |  | Three-band (pan-pLDH) |  |  |  |  |  |  |  |  |  |  |  |  |  |  |
| 13 |  | Three-band (Pv-pLDH) |  | M |  |  |  | W^a^ |  | W/F |  | M |  | ND |  | ND |
| 14 |  | Four-band (Pv-pLDH, pan-pLDH) |  |  |  |  |  |  |  | W |  |  |  | M^b^ |  |  |
| 15 |  | Four-band (Pv-pLDH, pan-pLDH) |  |  |  |  |  | M^b^ |  | W^a^ |  |  |  |  |  |  |
| 16 |  | Four-band (Pv-pLDH, pan-pLDH) |  |  |  |  |  | ND |  | W^a,b^ |  | M |  | ND |  | M^b^ |
| 17 |  | Four-band (Pv-pLDH, pan-pLDH) |  |  |  |  |  | ND |  | W^a^ |  |  |  | ND |  |  |
|  |  |  |  |  |  |  |  |  |  |  |  |  |  |  |  |  |
|  | Invalid: invalid test result: no appearance of a control line. | | | | | | | | | | | | | | | |
|  | ND: not performed because the sample or the Malaria RDT kit were exhausted. | | | | | | | | | | | | | | | |
|  | ^a^: test line intensity increased with 2 categories after dilution; in other cases, test line intensity increased with one category. | | | | | | | | | | | | | | | |
|  | ^b^: maximum dilution obtained at 50 × or 100 × dilution; in other cases maximum line intensity was obtained at 10 x dilution. | | | | | | | | | | | | | | | |
